# Supplementary material for: Deletion of wfs1 Impairs Oligodendrocyte Precursor Cells Dorsal Distribution and Myelination Through the wfs1-hmgcs1 Axis in Zebrafish
Source: Biology (Basel). 2026 Aug 16;15(16):1402. doi: 10.3390/biology15161402 (PMC13510151; doi:10.3390/biology15161402)
Supplement: Supplementary file 1 [file biology-15-01402-s001.zip › File S1-Transcriptome sequencing data and differentially expressed genes (DEGs) in wfs1-- zebrafish at 5 dpf/2.KEGG/wfs1KOvsWT/DOWN/src/dre04620.html]

dre04620


- dre:100002946

- Down regulated genes

100002946(-1.40391573648291)

- dre:792699

- Down regulated genes

792699(-1.84389282152821)

- dre:368481

- Down regulated genes

368481(-2.13889418398421)

- dre:403131

- Down regulated genes

403131(-4.42862381698358)

- dre:561021

- Down regulated genes

561021\_1(-4.68369619674599)

- dre:100000252

- Down regulated genes

100000252\_1(-1.7718817168434)

Close
